# From humans to the wild: revisiting assumptions in gut microbiome analysis

**DOI:** 10.1093/ismeco/ycag168

**Published:** 2026-06-15

**Authors:** Antton Alberdi, Ostaizka Aizpurua

**Affiliations:** Center for Evolutionary Hologenomics, Globe Institute, University of Copenhagen, 1350 Copenhagen, Denmark; Center for Evolutionary Hologenomics, Globe Institute, University of Copenhagen, 1350 Copenhagen, Denmark

**Keywords:** bias, metagenomics, microbiota

Gut microbiome research has become central to biomedical, agricultural, and eco-evolutionary sciences because of its relevance to understanding animal biology. From humans to mice and from chickens to newts, the communities of microorganisms inhabiting our guts are being investigated to uncover how microbial communities shape host physiology, health, and evolution. With high-resolution molecular methods and powerful computational tools, researchers can now reconstruct microbial genomes, infer gene functions, quantify expression patterns, and map the metabolites that define host–microbe interactions.

Despite all these advances, we argue that the field has not yet fully recognized the extent to which technical and biological differences across study systems influence gut microbiome analyses and their interpretation. Human-optimized frameworks implicitly set expectations for microbial DNA fraction, diversity recovery, genome reconstruction, and taxonomic assignability. Yet, the fully standardized analysis of over 3000 samples from more than 200 species within the Earth Hologenome Initiative recently surfaced the heterogeneity of vertebrate faecal samples regarding microbiome analyses [1]. From humans to wild hosts, disparities in prior knowledge, sample accessibility, and microbiome biology are substantial, demanding critical evaluation of whether tools and strategies developed in one context—usually the human one—can be meaningfully extrapolated without distorting downstream ecological interpretation [2].

These concerns become clear when considering the genomic resources that underpin much of microbiome research. Public databases continue to expand rapidly with the addition of new bacterial and archaeal genomes, yet their taxonomic and host breadth remains comparatively narrow. Most human-associated prokaryotes are now represented [3], but metagenomic genome reconstructions in livestock still yield discovery rates of 15%–30% (under a 5% average nucleotide identity species delimitation criterion), and surveys of wild vertebrates uncover up to 95% previously unrecorded species (Fig. 1a) [1]. Single microbial species can also show deep divergence between humans and other hosts, with significant functional differences across strains [4]. These patterns indicate that, in non-human systems, mapping shotgun sequencing reads to existing references or relying on amplicon sequencing of marker genes is likely to yield an incomplete and biased representation of gut microbiomes. Because many microorganisms still lack high-level taxonomic assignments, the ability to identify similarities and differences across studies is also sharply constrained. These limitations underscore the need for system-specific genome catalogues that capture the diversity present in each host.

**Figure 1:**
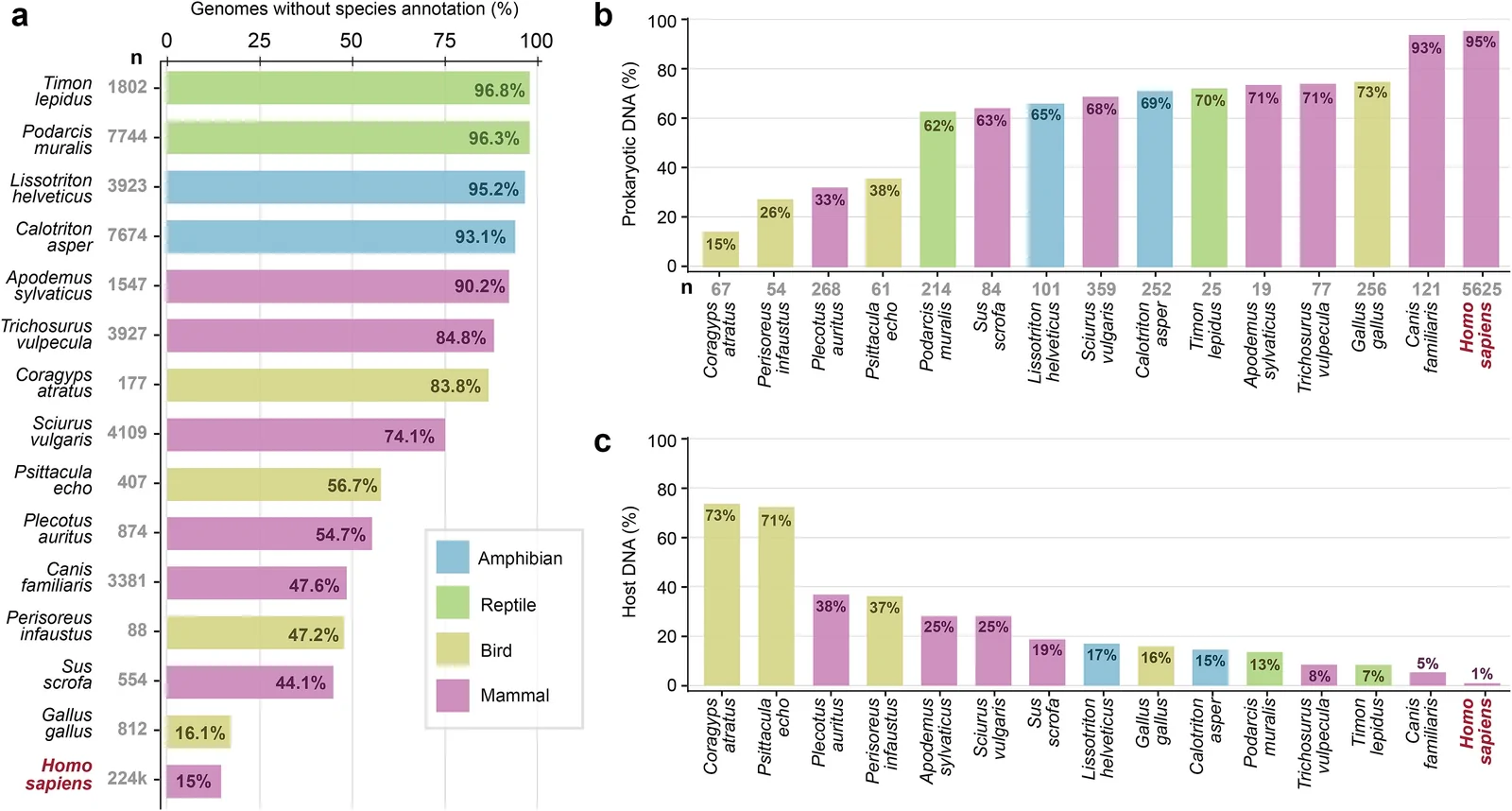
Quantitative metrics illustrating humans as an edge case in gut microbiomes across vertebrates. Host species were selected based on sample representation within each vertebrate clade in the Earth Hologenome Initiative datasets, with the most densely sampled species included to maximize comparability. Wild animal samples were processed using harmonized DNA extraction, sequencing, and quality-control protocols, and metagenomic data were analysed using standardized bioinformatic pipelines designed for cross-host comparability. Human results are drawn from Ma et al. (2026). (a) Species-level taxonomic assignment of metagenome-assembled genomes (MAGs), calculated as the fraction of MAGs with an average nucleotide identity within 5% of any genome available in the R220 release of the Genome Taxonomy Database (GTDB), containing reference genomes of 113 104 bacterial and archaeal species. N indicates the number of MAGs analysed per host taxon. (b) Bacterial and archaeal DNA fractions estimated using the SingleM prokaryotic fraction tool. *N* indicates the number of samples analysed per host taxon. (c) Host DNA fractions measured after mapping reads to host reference genomes. Note that some taxa lack reference genomes, which may lead to underestimation of their host DNA fractions. All estimations are based on faecal samples, except for *Gallus gallus* (caecum contents) and *Coragyps atratus* (colon contents).

However, the construction of such catalogues through metagenomic assembly and binning in non-model hosts is constrained by system-specific heterogeneity. Across vertebrates, gut structure varies widely in length, compartmentalization, retention time, epithelial specialization, and physicochemical conditions, making the human gastrointestinal tract a poor template for the diversity of digestive systems encountered in wild hosts [5]. One clear consequence of this diversity is that microbial density differs far more widely among wild vertebrates than among humans and laboratory mice, leading to broad differences in the ratio of prokaryotic to non-prokaryotic DNA. Whereas some terrestrial mammals carry high prokaryotic fractions (>90%) similar to humans, faecal samples from many birds and bats often contain low (mean < 40%) and highly variable (1%–99%) microbial loads (Fig. 1b) [6]. The distinct chemical properties of faecal material from ammonotelic (many fish and tadpoles), ureotelic (mammals and adult amphibians), and uricotelic (reptiles and birds) hosts can also affect DNA extraction success, further compounding the challenges of studying gut microbiomes across non-human taxa. Such conditions reduce sequencing efficiency and hinder comparable representations of microbial communities across species and individuals. When non-microbial DNA is host-derived and a reference genome is available, sequencing depth can be adjusted according to the measurable non-host fraction, although in some cases the overwhelming dominance of host DNA can make the required sequencing depth impractical (Fig. 1c). When the excess non-prokaryotic DNA originates from diet or environmental sources, assembly and binning performance degrade substantially.

These caveats obscure microbial signals, producing incomplete or biased genome catalogues and distorting downstream compositional and diversity estimates. Heterogeneity also extends beyond molecular properties of samples to the biological context of sample acquisition: hosts differ markedly in faecal form, defecation frequency, gut passage time, and accessibility of fresh material. These factors complicate standardization across taxa and limit the representativeness and comparability of resulting datasets. Because neither molecular nor biological homogeneity can be assumed, studies must scrutinize processing steps such as extracted DNA quantities and library preparation efficiency to determine whether variation in diversity and composition reflects true biology or technical artifacts.

The prokaryotic genomes recovered through metagenomic assembly and binning provide the basis for downstream functional annotation and for inferring the metabolic and ecological properties of gut microbiome members. However, functional annotation remains constrained by biases in widely used reference databases such as KEGG, COG, EggNOG, and CAZy, which are built primarily from cultured, human-associated model organisms. As a result, many ecologically relevant pathways present in non-human systems may remain undetected, misclassified, or difficult to interpret [7].

Interpretive bias, therefore, arises not only from technical limitations but also from conceptual inertia: the persistence of human-centred assumptions about diversity, dysbiosis, pathogen status, and host specificity as default reference points for microbiome research. For example, although in Western human adult populations, low microbiome diversity has been linked to dysbiosis and disease [8], this relationship does not generalize across other host species. Many non-human vertebrates show broad natural variation in microbiome diversity, and in some systems, disease states are linked to higher, rather than lower, diversity [9]. Likewise, taxa commonly viewed as human pathogens, such as *Fusobacterium*, are widespread, normal constituents of the gut microbiomes of many carnivorous and scavenging species [10].

All these differences argue for a reassessment of the core technical and biological assumptions that guide gut microbiome research beyond humans, particularly in wild hosts (Fig. 2). To obtain representative and unbiased results in non-human systems, criteria and thresholds derived from human studies must be reassessed and recalibrated across host contexts, rather than assumed to generalize across vertebrates. This requires benchmarking technical assumptions about sequencing depth, prokaryotic fractions, genome recovery, gene prediction, pathway-database coverage, and annotation reliability to separate true biological differences from analytical variation, while also revisiting interpretive assumptions about diversity, dysbiosis, pathogen status, host specificity, and the existence of broadly conserved core microbiomes. We therefore argue for a shift from treating human gut microbiomes as an implicit baseline toward treating host-specific microbiome properties as hypotheses to be tested across ecological and evolutionary contexts.

**Figure 2:**
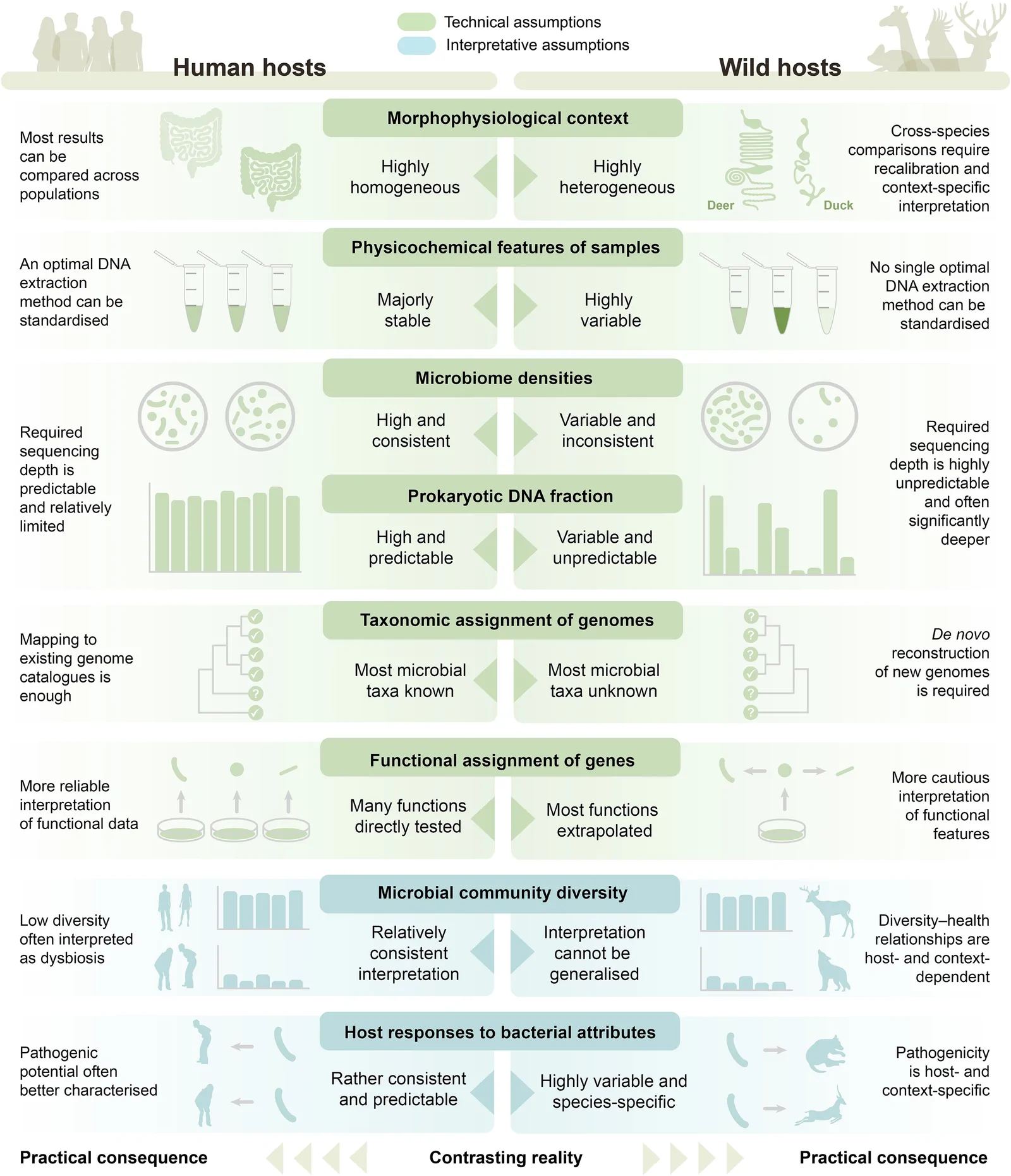
Conceptual shift required when moving from human-centred gut microbiome analysis to wild-host microbiome research.

Addressing this challenge will require coordinated action: expanding the representation of non-human microbial genomes in public databases; improving functional annotation frameworks to capture pathways relevant beyond humans; developing cross-species standards for sampling, metadata, and analytical pipelines; and explicitly testing the assumptions that underlie common diversity and compositional metrics. Such steps will help ensure that gut microbiome research reflects, rather than obscures, the diversity and complexity of hosts beyond humans, improving the quality of wild-animal microbiome studies while also providing the broader comparative context needed to interpret human gut microbiomes more accurately.
